# The Impact of a Gamified Intervention on Daily Steps in Real-Life Conditions: Retrospective Analysis of 4800 Individuals

**DOI:** 10.2196/47116

**Published:** 2024-08-12

**Authors:** Alexandre Mazéas, Cyril Forestier, Guillaume Harel, Martine Duclos, Aïna Chalabaev

**Affiliations:** 1 Laboratoire Sport et Environnement Social (SENS) Université Grenoble Alpes Grenoble France; 2 National Research Institute for Agriculture, Food and Environment (INRAE) Clermont-Ferrand France; 3 Kiplin Nantes France; 4 Laboratoire Motricité, Interactions, Performance (MIP - UR4334) Nantes Université Nantes France; 5 Department of Sport Medicine and Functional Exploration University Hospital Clermont-Ferrand, Hospital G. Montpied Clermont-Ferrand France

**Keywords:** behavior change, daily steps, exercise, gamification, intervention, mHealth, mobile health, mobile phone, physical activity, real world data, retrospective, self-determination theory, step, steps

## Abstract

**Background:**

Digital interventions integrating gamification features hold promise to promote daily steps. However, results regarding the effectiveness of this type of intervention are heterogeneous and not yet confirmed in real-life contexts.

**Objective:**

This study aims to examine the effectiveness of a gamified intervention and its potential moderators in a large sample using real-world data. Specifically, we tested (1) whether a gamified intervention enhanced daily steps during the intervention and follow-up periods compared to baseline, (2) whether this enhancement was higher in participants in the intervention than in nonparticipants, and (3) what participant characteristics or intervention parameters moderated the effect of the program.

**Methods:**

Data from 4819 individuals who registered for a mobile health Kiplin program between 2019 and 2022 were retrospectively analyzed. In this intervention, participants could take part in one or several games in which their daily step count was tracked, allowing individuals to play with their overall activity. Nonparticipants were people who registered for the program but did not take part in the intervention and were considered as a control group. Daily step counts were measured via accelerometers embedded in either commercial wearables or smartphones of the participants. Exposure to the intervention, the intervention content, and participants’ characteristics were included in multilevel models to test the study objectives.

**Results:**

Participants in the intervention group demonstrated a significantly greater increase in mean daily steps from baseline than nonparticipants (*P*<.001). However, intervention effectiveness depended on participants’ initial physical activity. The daily steps of participants with <7500 baseline daily steps significantly improved from baseline both during the Kiplin intervention (+3291 daily steps) and the follow-up period (+945 daily steps), whereas participants with a higher baseline had no improvement or significant decreases in daily steps after the intervention. Age (*P*<.001) and exposure (*P*<.001) positively moderated the intervention effect.

**Conclusions:**

In real-world settings and among a large sample, the Kiplin intervention was significantly effective in increasing the daily steps of participants from baseline during intervention and follow-up periods compared to nonparticipants. Interestingly, responses to the intervention differed based on participants’ initial steps, with the existence of a plateau effect. Drawing on the insights of self-determination theory, we can assume that the effect of gamification could depend of the initial motivation and activity of participants.

## Introduction

### Background

Physically inactive individuals are at higher risk of developing noncommunicable diseases—such as cardiovascular diseases, cancers, type 2 diabetes mellitus, or obesity—and mental health issues than those who are most active [1]. However, one-third of the world’s population is insufficiently active [2,3], and the trend is downward, with adults performing on average 1000 fewer steps per day than 2 decades ago [4]. In addition, it has recently been reported that the global population step count did not return to prepandemic levels in the 2 years following the onset of the COVID-19 outbreak [5]. The number of steps per day is a simple and convenient measure of physical activity (PA). Recent research suggests that an increase in the daily step count is associated with a progressively lower risk of all-cause mortality. Walking an additional 1000 steps per day can help reduce the risk of all-cause mortality [6]. For adults aged ≥60 years, this reduction in mortality rates is observed with up to approximately 6000 to 8000 steps per day, whereas for adults aged <60 years, the threshold is approximately 8000 to 10,000 steps per day [7]. However, sustaining this increase over time is crucial to achieve tangible health benefits [8]. Despite the efficacy of current programs in eliciting initial changes in individuals’ PA, they often struggle with inducing long-term behavioral shifts [9]. In this context, there is an urgent need to sustainably increase the number of daily steps of individuals in primary, secondary, and tertiary prevention.

Digital behavior change interventions are promising avenues to promote daily steps. Smartphones and digital tools, ubiquitous in our daily lives, offer several advantages, including their widespread availability, relatively low cost, and ability to access content quickly from anywhere [10-12]. Moreover, these technologies can collect real-time data in natural contexts (ie, daily step counts can be measured via accelerometers embedded in either commercial wearables such as Fitbit or smartphones) and present them in quantified formats, providing opportunities for exploration and reflection. This facilitates the implementation of powerful behavior change techniques such as goal setting and self-monitoring, potentially influencing behaviors [11]. However, there are concerns about the ability of digital programs to engage participants once the novelty wears off or to be effective on any type of audience regardless of their age, sociodemographic characteristics, or health status. In this context, gamification strategies introduce an exciting road map for addressing these challenges.

Gamification refers to the use of game elements in nongame contexts [13] and allows for the transformation of a routine activity into a more engaging one. Self-determination theory (SDT) [14] is a commonly used theoretical framework for understanding the motivational impact of gamification on behavior. SDT suggests the existence of different types of motivation that can be pictured on a continuum ranging from lack of motivation to completely autonomous motivation in which the behavior comes from the individual’s will. By contrast, controlled motivation will lead the individual to practice for the consequences that the activity can bring and not for the activity itself. SDT holds that people will be more likely to perform the behavior in the long term when their motivation is autonomous rather than controlled. Thus, autonomous forms of motivation represent more sustainable drivers of engagement and are an important predictor of the long-term maintenance of physical practice [15,16]. Autonomous motivation occurs when people perform an activity for their own satisfaction, inherent interest, and enjoyment. Moreover, 3 basic psychological needs are presumed to achieve self-determination: the need for autonomy (ie, need to feel responsible for one’s own actions), competence (ie, need to feel effective in one’s interactions with the environment), and relatedness (ie, need to feel connected to other people).

In addition to providing fun and playful experiences to users, gamification can effectively address basic psychological needs [17]. First, gamification strategies such as point scoring, badges, levels, and competitions serve to sustain the need for competence by offering feedback on users’ behaviors. Second, customizable game environments or user choices can support autonomy. Finally, features such as leaderboards, team structures, groups, or communication functions can foster a sense of relatedness. From this perspective, a gamified intervention would feed the autonomous motivation of participants and would be more correlated with the long-term adherence to PA. However, from another perspective, several criticisms have been leveled at gamification, including the fact that these mechanisms are reward oriented and that, still in line with SDT, the use of external rewards can reduce autonomous motivation [18,19].

A recent meta-analysis [20] revealed that digital gamified interventions lasting on average 12 weeks improved daily steps by 1600 steps on average. Importantly, the results showed that gamified interventions (1) appear more effective than digital nongamified interventions, (2) seem appropriate for any type of user regardless of their age or health status, and (3) lead to a persistent PA improvement after follow-up periods lasting on average 14 weeks with a very small to small effect size. As a result, gamified interventions are emerging as interesting behavior change tools to tackle the physical inactivity pandemic. However, these findings obtained from randomized controlled trials do not always reflect what happens in real-life settings [21]. In addition, the effect sizes reported in this meta-analysis were heterogeneous, and the authors found high between-study heterogeneity (eg, *I*^2^=82%).

If this heterogeneity can be explained by differences in study quality or diversity of designs in the included studies, the behavior change intervention ontology proposed by Michie et al [22] argues that heterogeneity in behavioral interventions could also be explained by different variables such as intervention characteristics (eg, content and delivery), the context (eg, characteristics of the population targeted, such as demographics, and setting, such as the policy environment or physical location), exposure of participants to the program (eg, engagement and reach), and the mechanisms of action (the processes through which interventions influence the target behavior). Considering these variables within gamification contexts could provide a useful means to better understand the conditions under which interventions are successful. Furthermore, based on SDT, we can envisage that gamification techniques will not have the same impact on all users depending on their initial motivation and the way they perceive games.

This study investigated these questions based on a retrospective analysis of real-world data collected from a large sample of adult participants who were proposed a mobile health gamified intervention developed by the company Kiplin in France from 2019 to 2022. In this intervention, participants could take part in one or several collective games in which their daily step count was tracked, allowing individuals to play with their overall activity. In addition to offering the possibility of direct intervention on people’s activity habits in a natural context, the capacity of this mobile app to collect a large amount of objective real-world data in real time can be useful for understanding the processes and outcomes of behavioral health interventions [23]. More specifically, these data can help make explicit when, where, for whom, and in what state for the participant the intervention will produce the expected effect, notably owing to continuous data collection over time. The within-person evolution in daily steps obtained via the app combined with between-person individual factors and intervention parameters is of great interest in this perspective.

### Objectives

Thus, the objectives of this study were to analyze the data collected to (1) examine within-individual evolutions of daily steps before, during, and after the intervention; (2) test the effectiveness of a gamified program in real-life conditions on daily steps among participants versus nonparticipants; and (3) explore the variables that could explain heterogeneity in responses to the intervention. On the basis of previous results on gamification [20], we first hypothesized that daily steps would increase during and after the gamified program compared to baseline (hypothesis 1). Second, we hypothesized that this improvement will be greater for participants than for nonparticipants (ie, participants who registered on the app but did not complete any games; hypothesis 2). Finally, we expected that the intervention’s characteristics (ie, type and number of games), the context within which the intervention was performed (ie, population and setting), and the exposure to the intervention (ie, engagement of participants with the app) will moderate the intervention effect (hypothesis 3).

## Methods

### Study Design and Participants

**Figure 1:**
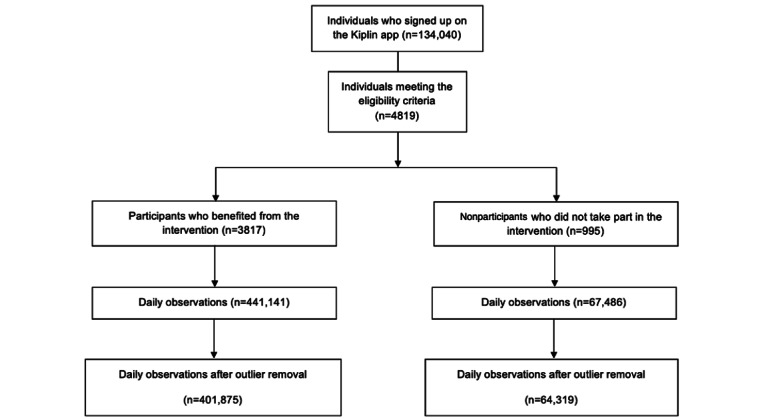
Study flowchart and screening of the Kiplin database.

This study retrospectively analyzed data from adult participants who had registered for a Kiplin program and had given consent for their data to be collected. To be included, participants must be aged ≥18 years; have registered on the app between January 1, 2019, and January 2, 2022; and logged daily steps (measured via their smartphone or an activity monitor) on a time frame of at least 90 days with <20% of missing daily observations. Of the 134,040 individuals who registered on the Kiplin app during this time span, 4819 (3.6%) met the eligibility criteria. Figure 1 shows the study flowchart.

Nonwear days were defined as days with <1000 steps and considered as missing observations—as previous research has suggested that daily step values of <1000 may not represent full data capture [24,25]. Days before the first day of the first game were considered as *baseline* (median 14, SD 42.9 days), the period between the first day of the first game and the last day of the last game was considered as the *intervention period* (median 19, SD 31.2 days), and the days after the last day of the last game were considered as the *follow-up* (median 90, SD 22.8 days). We restricted the follow-up periods to 90 days after the intervention (ie, 3 months).

Participants could receive the Kiplin intervention (1) in the context of their work (ie, primary prevention with employees), (2) in an older adult program (ie, primary prevention with volunteer retirees), or (3) as part of their chronic disease care (ie, patients mainly treated for obesity or cancer). In all the aforementioned conditions, the program was paid not by the participant but by their employer or health care center.

Some participants registered for the program and created an account but did not take part in the intervention (ie, did not complete any games). These individuals were considered *nonparticipants* and were used as a control group (as proposed in previous research [26]). Similarly, the baseline period for these nonparticipants corresponded to the days before the date in which they were supposed to start the intervention period.

### Ethical Considerations

This study was approved by the local ethics committee (IRB00013412; CHU de Clermont-Ferrand institutional review board 1; institutional review board number 2022-CF063) with compliance with the French policy of individual data protection.

### The Kiplin Intervention

The Kiplin intervention proposes time-efficient collective games accessible through an Android or iOS app. In all games, participants’ daily step counts are converted into points, allowing for progression within the games. The Kiplin app retrieves participants’ daily step counts by integrating with the application programming interfaces (APIs) of the apps used by participants to track their activity (such as Apple Health for iPhone users, Google Health for Android users, and Garmin Health). In this way, participants could connect a wearable if they already owned one. In addition, participants had access to a visual tool to monitor their daily and weekly step counts and to a chat for communication with other participants. Depending on the program, participants were offered one or several games lasting approximately 14 days each. If several games were proposed, these games followed each other in an interval of <60 days.

**Figure 2:**
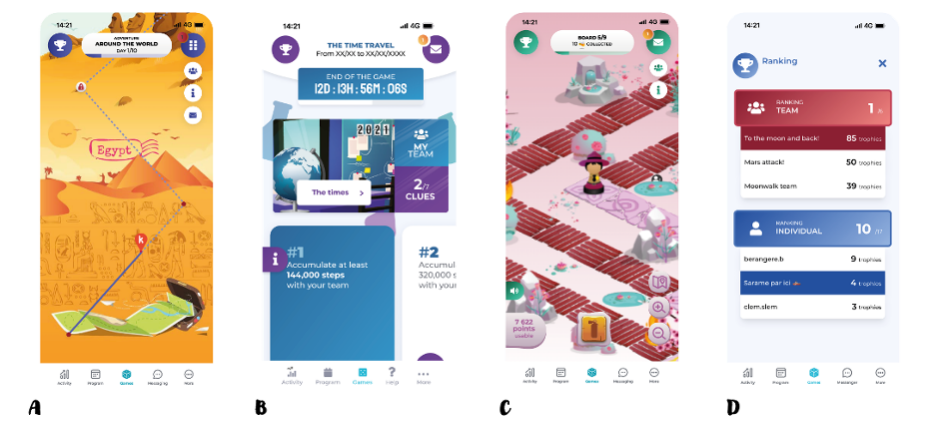
Screenshots of the Kiplin games. (A) The Adventure. (B) The Mission. (C) The Board Game. (D) The Challenge.

Participants could take part in 4 different games with no option for selection. In The Adventure, the objective was to reach step goals collectively to progress toward a final destination. Players could track their progress on a map, with checkpoints representing distances between different cities of a digital world tour (Figure 2A). In The Mission, participants engaged in PA and collective challenges to unlock clues and attempt to solve missions (Figure 2B). In The Board Game, participants took on the role of forest rangers tasked with extinguishing fires. Achieving step goals allowed for progress on the board and advancement to higher levels, ultimately aiming to extinguish all fires and save forest residents (Figure 2C). Finally, in The Challenge, players aimed to achieve the highest number of steps and complete challenges to earn trophies for their team. Team and individual rankings were available (Figure 2D).

These games included a multitude of gamification mechanisms such as points, trophies, leaderboards, a chat, challenges, and narratives—mechanics that are closely linked to proven behavior change techniques [27]. Table 1 gives an overview of the gamification strategies included in the Kiplin games following the taxonomy proposed by Schmidt-Kraepelin et al [28] and the associated behavior change techniques. While the games share common characteristics (eg, collective gameplay and in-game challenges), it is important to note that The Adventure and The Challenge emphasize competition more than the others.

**Table 1:** Analysis of the game mechanics and content of the Kiplin app and associated behavior change techniques following the taxonomies by Schmidt-Kraepelin et al [28] and Michie et al [27].

Dimension	Characteristic	Gamification technique	Associated behavior change techniques
Gamification concept-to-user communication	Mediated	The gamification concept communicates with the user through an avatar named Pilot Kiplin (ie, a real Kiplin team member animating the app who takes the persona of a funny mascot). Pilot Kiplin launches in-game challenges, announces results, and delivers internal messages aimed at motivating participants. These messages include tips to plan and implement PA^a^ in daily life and information on the benefits of walking on health.	Instruction on how to perform a behavior (4.1) Information about health consequences (5.1)
User identity	Static self-selected identity	Participants are only able to select a nickname and personalize the name of their team.	—^b^
Rewards	Internal	Rewards that participants can earn through the app are solely internal and virtual, such as points and trophies.	Feedback on behavior (2.2) Self-monitoring of behavior (2.3) Cue signaling reward (7.2) Behavioral practice and rehearsal (8.1) Nonspecific reward (10.3)
Competition	Direct and indirect	Participants can compete directly via in-game challenges (ie, at specific intervals, a competitive challenge commences between teams; to secure victory and earn a trophy, one team must accumulate more points or steps than their rival team within the allotted time frame) or indirectly via the point system and leaderboards. These challenges are announced in advance, encouraging players to plan their activities so as to be active on the day of the battle.	Action planning (1.4) Feedback on behavior (2.2) Social comparison (6.2)
Target group	Patients and healthy individuals	The app can be offered to patients in clinical settings as well as in preventive initiatives among employees or older adults.	—
Collaboration	Cooperative	All games operate on a collaborative basis: participants are organized into teams and must collectively complete challenges to advance or win. They can use the chat feature to communicate, support each other, and exchange ideas with their teammates.	Social support (unspecified, 3.1) Social support (emotional, 3.3)
Goal setting	Externally set	Goals, cutoffs, and the number of steps required to earn a trophy are predetermined by the app developer or the health care professional.	Review behavior goals (1.5) Discrepancy between current behavior and goal (1.6)
Narrative	Episodical	Narratives are unlocked as participants reach new milestones, such as unlocking a new clue in The Mission, reaching a higher board level in The Board Game, or reaching a new city in The Adventure.	—
Reinforcement	Positive	The app focuses solely on highlighting current and future successes, aiming to avoid stigmatizing users based on their initial inactivity or health status.	—
Persuasive intent	Behavior change	The app aims to enhance individuals’ daily step counts through game mechanics.	—
Level of integration	Inherent	To participate in the games, individuals must engage in PA. Progression within the game is contingent upon the steps performed; without activity, players cannot advance.	—
User advancement	Presentation only	The progression is presented through the clues collected in The Mission to the progression to different cities in The Adventure and to new board levels in The Board Game. This structure encourages participants to engage in and repat the target behavior.	Graded tasks (8.7) Reward approximation (14.4)
Other tools of the app	—	Self-monitoring tool and notifications	Self-monitoring (2.2) Prompt and cues (7.1)

^a^PA: physical activity.

^b^No correspondence with the Behavior Change Technique Taxonomy.

### Variables

The variables of interest were selected based on the behavior change intervention ontology by Michie et al [22] and included (1) the longitudinal evolution of daily steps, (2) the exposure of each participant to the intervention, (3) the intervention parameters, and (4) the context (participants’ characteristics and setting) as these variables are likely to influence the intervention effect. Table 2 specifies the measures of interest and their operationalization.

**Table 2:** Operationalization of the variables.

Outcome			Operationalization
**Primary outcome—target behavior (dependent variable)**			
	Daily step count	PA^a^ was assessed via the daily step count, measured using the smartphone or activity monitor of the participant. The daily step count is a trusted proxy for PA [29]. During onboarding, participants were asked to connect to their tracking device (eg, Apple Health, Google Fit, Fitbit, or Garmin) for synchronization of their step count data. In this way, the daily step count of the participants was automatically synchronized on the Kiplin app, and the app could retrieve the daily step count for the previous 15 days.	
**Intervention (content and delivery) and mechanisms of action**			
	Type of game	Participants could play 4 types of games (ie, The Challenge, The Adventure, The Board Game, and The Mission).	
**Exposure**			
	Compliance ratio	The engagement of participants with the app was computed as the compliance ratio representing the number of days with a log-in during the game period divided by the duration of the game periods. This variable allows for measuring the frequency of the engagement with the service [30].	
	Number of games played	The total number of games played during the intervention period	
**Context (population and setting)**			
	Self-reported age and gender	Filled out by participants when they registered on the app	
	Population	Employees, older adults, or patients (treated for obesity or cancer)	
**Confounding factors**			
	Season	The season (winter, spring, summer, or autumn) when the step data were logged was controlled for as the season can influence PA [31].	
	Type of device	The type of device used to assess daily step count (ie, Android or iOS smartphones or Garmin, Withings, Polar, Fitbit, or TomTom wearables) was controlled for as smartphone apps and wearable devices differ in accuracy and precision [32].	
	Lockdown	The study period was characterized by the COVID-19 pandemic. In France, 3 lockdowns were implemented to mitigate the spread of COVID-19: in spring 2020 from March 17 to May 11, in fall 2020 from October 30 to December 15, and in spring 2021 from April 3 to May 3. During these periods, French citizens were required to remain at home with exceptions for essential activities such as going to work, shopping for necessities, health purposes, and engaging in individual PA near their residence. Failure to provide documentation justifying outdoor movement during inspections could result in fines. As these periods had a strong influence on the PA of individuals [33], we controlled for the lockdown periods in our analyses.	

^a^PA: physical activity.

### Statistical Analyses

We calculated the step count increase by subtracting the baseline average daily step count from the average daily step count during the intervention or follow-up periods for each participant and then computed the relative change (in percentage).

Mixed-effects models were used to (1) analyze within-person evolution across time (ie, changes in daily steps throughout the baseline, intervention, and follow-up periods) and across participants and nonparticipants and (2) examine the associations among intervention parameters, exposure to the intervention, participants’ characteristics and settings, and daily step evolution. This statistical approach controls for the nested structure of the data (ie, multiple observations nested within participants); does not require an equal number of observations from all participants [34]; and separates between-person from within-person variance, providing unbiased estimates of the parameters [35,36].

First, an unconditional model (ie, with no predictor) was estimated for each variable to calculate intraclass correlation coefficients and estimate the amount of variance at the between- and within-individual levels, which allowed us to determine whether conducting multilevel models was relevant or not. Then, a model that allowed for random slope over time (ie, model with random intercept and random slope) was compared to the null model (ie, with only random intercept) using an ANOVA to evaluate whether the less parsimonious model explained a significantly higher proportion of the variance of the outcome than the unconditional model [37,38]. Third, between-level predictors and confounding variables were added to another model (model 1; the equation for the model was as follows: Y_ij_ = [β_0_ + γ_0i_ + θ_0j_] + [β_1_ + θ_1j_] time_j_ + β_2_ phase_j_ + β_3_ age_j_ + β_4_ sex_j_ + β_5_ population_j_ + β_6_ season_j_ + β_7_ captor_j_ + β_8_ baseline PA_j_ + β_9_ lockdown_j_ + β_10_ condition_j_ × phase_j_ + ε_ij_, where β_0_ to β_10_ are the fixed-effects coefficients, θ_0j_ and θ_1j_ are the random effect for participant *j* (1 random intercept and 1 random slope), γ_0i_ is the random effect for time *i* [random intercept], and ε_ij_ is the error term) and compared to the previous models. Finally, intervention characteristics, as well as their interactions with the phases (ie, baseline, intervention, or follow-up) of the study, were added in a final model excluding nonparticipants (model 2; the equation for the model was as follows: Y_ij_ = [β_0_ + γ_0i_ + θ_0j_] + [β_1_ + θ_1j_] time_j_ + β_2_ phase_j_ + β_3_ age_j_ × phase_j_ + β_4_ sex_j_ + β_5_ population_j_ × phase_j_ + β_6_ season_j_ + β_7_ captor_j_ × phase_j_ + β_8_ baseline PA_j_ × phase_j_ + β_9_ lockdown_j_ + β_10_ compliance ratio_j_ × phase_j_ + β_11_ number of games played_j_ × phase_j_ + β_12_ type of game_j_ + ε_ij_, where β_0_ to β_12_ are the fixed-effects coefficients, θ_0j_ and θ_1j_ are the random effect for participant *j* (1 random intercept and 1 random slope), γ_0i_ is the random effect for time *i* [random intercept], and ε_ij_ is the error term). Model fit was assessed via the Bayesian information criterion and –2 log-likelihood [39]. All models were performed using the *lmerTest* package in the R software (R Foundation for Statistical Computing) [40]. An estimate of the effect size was reported using the marginal and conditional pseudo-*R*^2^. When the interaction terms turned significant, contrast analyses were computed using the *emmeans* package [41]. The models’ reliability (estimated using residual analyses) and outlier detection were performed using the *Performance* package [42]. In addition to subtracting nonwear days (defined previously), we removed outliers via the *check_outliers* function [42] that checks for influential observations via several distance and clustering methods (ie, *Z* scores, IQR, and equal-tailed interval). Sensitivity analyses were conducted using all data (including data before outlier imputation) and are available in Multimedia Appendix 1.

The data and code for the statistical analyses used in this study are available on the Open Science Framework [43].

## Results

### Descriptive Results

Descriptive results are presented in Table 3. The final sample included 4819 adults (mean age 42.7, SD 11.5 y; 2823/4819, 58.58% women). Participants wore an activity monitor measuring their daily step count for an average of 113 (SD 58.01; range 90-686) days. A total of 34,922 daily step observations were missing (ie, daily data missing or considered as a nonwear day), which is equivalent to 6.4% of missing data for the full data set.

We tested for statistical differences in sociodemographic variables and baseline daily steps between participants and nonparticipants using 2-tailed *t* tests and chi-square tests. Results revealed significant differences for age (*t*_82,500_=–6.9149; *P*<.001), gender (*χ*^2^_2_=4028.3; *P*<.001), and baseline daily steps (*t*_22,721_=–19.75; *P*<.001). However, in large samples, *P* values may drop below the α level despite effect sizes that are not practically meaningful [44]. Therefore, we mainly examined the magnitude of the effect sizes of these differences and observed very small to small effects (*d*=–0.03 for age, *d*=–0.17 for baseline daily steps, and *w*=0.09 for gender). According to Magnusson [45], the interpretation of these effect sizes suggests that, for age and baseline daily steps, approximately 98.8% and 93.2% of individuals in both groups overlapped, respectively. In addition, there is approximately a 50.8% and 54.8% chance that a randomly selected individual from the nonparticipant group would have a higher score than a randomly selected individual from the participant group. Therefore, we considered that the differences were minor between the 2 groups. Finally, these variables were controlled in our mixed-effects models as they were included as fixed effects.

**Table 3:** Descriptive statistics.

		Participants (n=3817)	Nonparticipants (n=995)
**Sociodemographic characteristics**			
	Age (y), mean (SD)	43.2 (11.08)	41.0 (12.81)
	Female sex, n (%)	2313 (62.6)	510 (53.26)
	Employees, n (%)	3526 (92.38)	978 (98.29)
	Patients, n (%)	194 (5.16)	17 (2.09)
	Older adults, n (%)	97 (2.54)	—^a^
**Exposure, mean (SD)**			
	Compliance ratio	0.84 (0.23)	0 (0)
	Games played	1.28 (0.9)	0 (0)
	In-game days	22.06 (16.24)	0 (0)
**Observations in each type of game, n (%)**			
	The Adventure	21,316 (32.73)	—
	The Board Game	4093 (6.28)	—
	The Challenge	32,801 (50.37)	—
	The Mission	6915 (10.62)	—
**Type of device used, n (%)**			
	Android smartphone	1076 (28.19)	286 (28.74)
	iOS smartphone	810 (21.22)	533 (53.57)
	Fitbit	750 (19.65)	52 (5.23)
	Garmin	1071 (28.06)	109 (10.95)
	Polar	5 (0.08)	—
	TomTom	3 (0.08)	—
	Withings	90 (2.36)	9 (0.9)
**Observations in each season, n (%)**			
	Winter	110,517 (23.87)	17,451 (24.4)
	Spring	94,961 (20.51)	21,162 (29.6)
	Summer	129,039 (27.87)	8804 (12.31)
	Fall	138,429 (29.9)	24,086 (33.67)
**Observations in each lockdown, n (%)**			
	First lockdown (spring 2020)	10,872 (2.35)	925 (1.29)
	Second lockdown (fall 2020)	32,298 (6.89)	4110 (5.75)
	Third lockdown (spring 2021)	23,435 (5.06)	1757 (2.46)

^a^Not applicable.

### Hypothesis 1: Is the Gamified Program Effective to Promote PA?

During the intervention period, participants increased their daily steps by 2619 steps per day on average (+55.6%) compared to the baseline period and by 317 steps per day on average during the follow-up period (+13.8%) compared to the baseline. In comparison, the daily step count of the control group remained more or less stable throughout the same time frame, with a mean increase of 151 daily steps compared to baseline (+7.5%).

**Figure 3:**
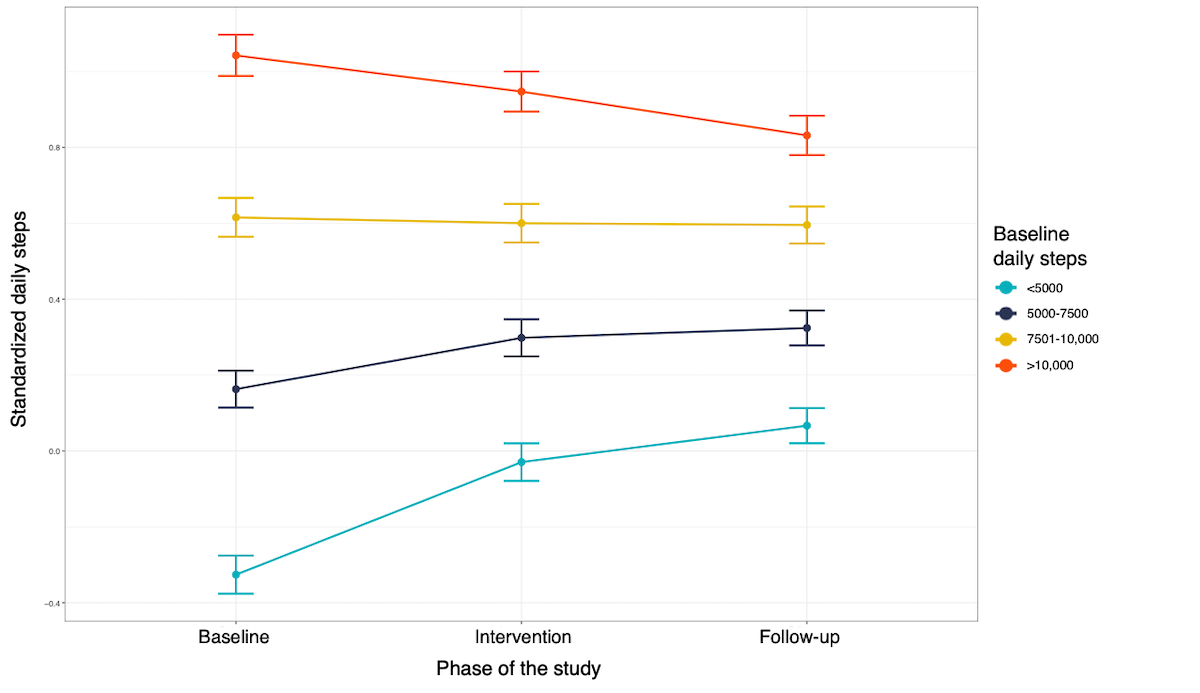
Changes in daily steps throughout the study phases for participants who received the Kiplin program stratified by baseline physical activity.

Overall, contrast analyses of the model for the intervention participants (model 2; Table S1 in Multimedia Appendix 1) revealed a negative effect of the intervention on the daily step count during the intervention phase compared to baseline activity (*b*=–0.09, 95% CI –0.14 to –0.05; *P*<.001) and no significant effect (*b*=0.01, 95% CI –0.05 to 0.06; *P*=.79) during the follow-up periods compared to baseline. However, the patterns were different when participants were stratified by baseline PA. Participants with lower baseline daily steps (<5000 steps per day or 5001-7500 steps per day) showed a significant increase in their daily steps during the intervention (*b*=0.25, 95% CI 0.22-0.28; *P*<.001) and follow-up (*b*=0.12, 95% CI 0.09-0.15; *P*<.001) periods both compared to the baseline. Participants with initial values between 7501 and 10,000 steps did not have a significant increase in their daily steps during the intervention (*b*=0.00, 95% CI –0.05 to 0.05; *P*=.99) or during the follow-up period (*b*=–0.01, 95% CI –0.04 to 0.02; *P*=.44) compared to baseline. Participants who performed >10,000 baseline steps had significant deteriorations during the intervention (*b*=–0.13, 95% CI –0.19 to –0.08; *P*<.001) and follow-up (*b*=–0.06, 95% CI –0.10 to –0.03; *P*<.001) periods. These trends are depicted in Figure 3 and Table 4. Results were similar in sensitivity analyses that used data without outlier imputation except for participants with initial daily step counts between 7501 and 10,000, who showed significant improvements during and after the intervention (Tables S2 and S3 in Multimedia Appendix 1).

In parallel, contrast analyses comparing the effectiveness of the Kiplin intervention on participants who used smartphones to collect their daily steps in comparison to participants who used a wearable showed a significantly greater effect among smartphone users during both the intervention phase (*b*=0.09, 95% CI 0.07-0.11; *P*<.001) and the follow-up period (*b*=0.04, 95% CI 0.01-0.06; *P*=.001). These results are illustrated in Figure S1 in Multimedia Appendix 1.

**Table 4:** Description of the mean daily step count during the baseline, intervention, and follow-up periods; changes; and relative changes from baseline according to participants’ baseline daily step count.

	Participants with <5000 steps	Participants with 5000-7500 steps	Participants with 7501-10,000 steps	Participants with >10,000 steps
Baseline daily step count, mean (SD)	3671 (902.73)	6096 (747.49)	8818 (824.84)	10,111 (1789.2)
Intervention daily step count, mean (SD)	7490 (3804.69)	8855 (3786.71)	10,301 (3627.73)	11,388 (3518.07)
Follow-up daily step count, mean (SD)	5119 (2062.40)	6534 (1889.99)	7971 (2074.43)	9424 (2390.33)
Change from baseline during the intervention	+3820	+2762	+2187	+1309
Change from baseline during follow-up	+1459	+431	–156	–697
Relative change during intervention (%)	+118.8	+47.2	+28.8	+16.9
Relative change during follow-up (%)	+49.5	+8.2	–1	–4.3

### Hypothesis 2: Is the Intervention Effect Greater for Participants Than for Nonparticipants?

In model 1 (Table S1 in Multimedia Appendix 1), participants who received the Kiplin intervention had a significantly greater increase in mean daily steps between baseline and the intervention period compared with nonparticipants (*b*=0.54, 95% CI 0.52-0.58; *P*<.001). The results were similar in sensitivity analyses (Table S3 in Multimedia Appendix 1). The comparison of the means, changes, and relative changes from baseline for participants and nonparticipants are available in Table S4 of Multimedia Appendix 1.

### Hypothesis 3: What Are the Moderators of the Intervention Effect?

**Figure 4:**
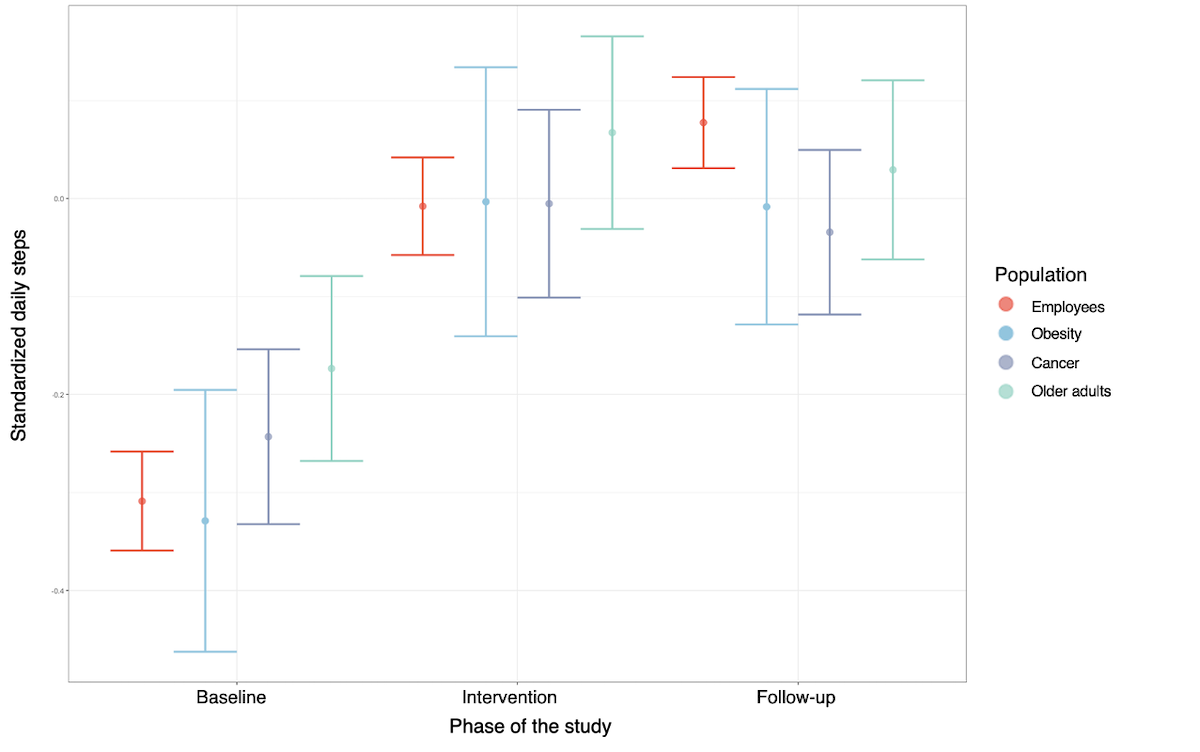
Changes in daily steps throughout the study phases for the different populations who received the Kiplin program.

The model 2 estimates are shown in Table S1 in Multimedia Appendix 1. The variables under consideration explained 39% of the variance in daily steps. In this model, we tested the hypothesized interactions to investigate predictors associated with the efficiency of the intervention (Table S5 in Multimedia Appendix 1). Contrast analyses were conducted on significant interactions and revealed that the age (*b*=0.05; *P*<.001) and compliance ratio (*b*=0.37; *P*<.001) were positively associated with the change in daily steps between baseline and the intervention period. Specifically, the older the age, the more regularly the individuals played and the more effective the intervention was. On the other hand, the number of games played by participants was negatively associated with this change (*b*=–0.02; *P*=.02). In other words, the longer the intervention and the higher the number of games, the less effective the intervention. For categorical outcomes, contrast analyses revealed differences in the intervention effect among the different populations (Figure 4). Compared to employees, patients treated for cancer (*b*=–0.18; *P*<.001) and older adults (*b*=–0.19; *P*<.001) showed a significantly weaker effect of the intervention in comparison to baseline PA. There was no significant difference between employees and patients treated for obesity (*b*=–0.07; *P*=.13). All the results of these analyses are available in Multimedia Appendix 1.

Finally, model 2 estimates revealed that participants were significantly more active in The Adventure and The Challenge compared to The Board Game and The Mission (Table S1 in Multimedia Appendix 1).

## Discussion

### Principal Findings

This study demonstrated a significant increase in daily steps among participants engaging with the Kiplin intervention compared to nonparticipants over the same period. Interestingly, the intervention effect varied according to the baseline daily step count of individuals. Participants with lower baseline steps (<7500 steps per day) significantly improved their PA during both the intervention (between +34% and +76%) and follow-up (between +10% and +33%) periods, whereas participants with >7500 steps had no significant change or significant decreases.

These results suggest that a gamified program is more efficient for inactive individuals compared to active ones, with the existence of a plateau effect. They also support recent findings [20,46] and the ability of gamified interventions to improve daily steps both during and after the end of the program and in real-life settings [47]—at least for the more inactive individuals. This efficacy is noteworthy given the challenges faced by current behavioral interventions in promoting PA in the long haul [9].

SDT offers a valuable framework for elucidating the disparate outcomes observed among initially active and inactive participants. Gamification strategies could enhance the autonomous motivation of inactive participants, as suggested by a previous study [48], whereas the use of rewards on already motivated people could undermine this motivation. Known as the overjustification effect [18], this phenomenon suggests that, if people receive rewards for doing an activity that they used to enjoy, they are likely to discount the internal reason and, thus, become less intrinsically motivated than before receiving the rewards. This could explain why the same intervention had positive effects on inactive participants, who performed more daily steps after the end of the intervention (ie, during follow-up periods), compared to its effects on already active ones, who observed significant decreases after the intervention compared to their baseline daily steps.

Moreover, results indicating that the intervention was more effective among users who used their smartphones to track their step counts through the Kiplin app compared to those who already owned and used a wearable device—and were significantly more active at baseline—further reinforce this argument. Individuals who already possess an activity monitor are likely motivated to monitor their daily steps, potentially diminishing the additional impact of gamification rewards. Consequently, the introduction of gamification may have less influence or even produce counterproductive effects on their behavior, particularly when compared to those who solely rely on their smartphones for activity tracking in the context of the intervention.

The results of this study also stressed that older age may not be incompatible with gamified interventions. Indeed, intervention effectiveness was moderated by the age of the individual, and gamification was more efficient among older individuals compared to younger ones. These findings are in line with those of a previous study [49] that reported higher use of gamification features among older users. The authors postulated that older adults pay generally more attention to their health and, thus, have a stronger intention to engage in a health program. From another perspective, and in light of the gamification strategies embedded in the Kiplin intervention, these results could also be explained by the fact that these strategies are accessible—inspired by traditional board game rules and mechanics widely known in the general population—and, thus, may be more attractive for older populations. Previous research has suggested that the most engaging game mechanics may diverge between youths and other populations [50], and we can expect that younger populations may prefer more complex game mechanics and need more novelty during the intervention to stay interested in the service.

Regarding the effects of the gamified intervention according to the characteristics of the population, a stronger effect was found for programs among employees and patients treated for obesity. While these results warrant caution due to the variability observed in patients or older adult participants, these findings suggest that gamified interventions are suitable for both primary and tertiary prevention, as suggested by previous work [20].

### Practical Implications

The findings of this study also offer valuable insights that could help improve future intervention design. First, exposure to the content is essential for the gamified intervention to be effective. It is interesting, as gamification has often been assimilated into a self-fulfilling process permitting automatic engagement of participants. These results are consistent with previous findings demonstrating that higher use of gamification features was associated with greater intervention effectiveness [49,51]. If gamification can ultimately increase program engagement, developers need first to design their apps to be as attractive as possible and optimize retention.

Second, the results revealed that the total number of games played was negatively associated with the intervention effect, suggesting that shorter interventions could be more beneficial for behavior change. These results are in line with those of previous research [20,52] suggesting that digital interventions of <3 months tend to yield greater benefits. It also suggests a “dose-response” relationship in an inverted U shape, with an optimal “middle” to find. Nevertheless, it is important to consider that Kiplin programs incorporating multiple games are built in such a way as to administer several doses at regular intervals. Therefore, periods without games were considered in the intervention phases and could explain why, overall, the shorter games were more efficient. More refined analyses of the intervention effect over time will be necessary in the future.

Third, the daily step count of participants was significantly higher in The Adventure and The Challenge. These 2 games are characterized by their competitive nature, placing a stronger emphasis on leaderboards than the other 2 games, which are more centered on collaboration. In this vein, Patel et al [53] observed that the competitive version of their gamified intervention outperformed the collaborative and supportive arms. Moreover, various studies have highlighted that leaderboards are a particularly successful gamification mechanic [49,54].

### Strengths and Limitations

This study has several strengths, including its large sample size, the intensive objective PA measurement in real-life conditions through daily steps, and the longer baseline and follow-up duration compared with most trials on gamification that typically incorporate measurement bursts dispersed across time [20]. However, several limitations should be considered. First, this study was observational and not a randomized controlled trial. Thus, we cannot establish the causality of the intervention’s effect on outcome improvement. The nonparticipants are not a true control group. If they did not receive the intervention, it may be because they were unable to join or for underlying motivational reasons that could impact their PA. Second, intervention lengths differed between participants. Third, although mixed-effects models are useful for describing trends in PA behavior change over time, they are limited in their capacity to assess precise fluctuation patterns of nonstationary behavior, such as daily step counts [55] across time. Future longitudinal studies could benefit from using time-series analyses to more accurately describe these patterns of change. Finally, the compliance ratio used in this study as a proxy for engagement tends to oversimplify the exposure of participants to the service. Complementary measures of behavioral engagement (eg, using the number of log-ins, time spent per log-in, and the number of components accessed) and affective engagement (eg, emotions and pleasure) should be considered to draw the longitudinal impact of the engagement of the participants on the intervention effect.

### Conclusions

In this study, we conducted a comprehensive analysis of real-world data from >4800 individuals, suggesting the impact of a gamified intervention in real-life settings. Our findings indicate that the Kiplin intervention led to a significantly greater increase in mean daily steps from baseline among users than among nonparticipants. Interestingly, responses to the intervention were significantly different as a function of individuals’ initial daily step counts. Participants with <7500 baseline daily steps had significant improvements during both the intervention and follow-up periods with +3291 daily steps during the program and +945 after the intervention on average, whereas the intervention had no effect on participants with initial values of >7500. Therefore, the motivational effect of gamification could depend on the initial PA and motivational profile of the participants. This result can also be interpreted in light of our observation that participants who already owned a wearable and, thus, were likely already motivated to engage in PA exhibited significantly lower effects compared to less experienced participants who used their smartphones to track their step counts. This study also revealed that the age of participants and their engagement with the app were positively and significantly associated with the intervention effect, whereas the number of games played was negatively associated with it.

Overall, the results of this study suggest that gamification holds promise in promoting the daily steps of inactive populations, with demonstrated short- and medium-term effects. Importantly, this study represents a pioneering effort as one of the first to examine the longitudinal effect of a gamified program outside the context of a trial using intensive real-world data. As such, the findings are quite generalizable to similar settings and reaffirm the value of gamification in both primary and tertiary prevention efforts across a diverse range of age groups.

## Appendix

Multimedia Appendix 1 Supplementary Figure S1 and Table S1-S5.

## Abbreviations

API: application programming interface
PA: physical activity
SDT: self-determination theory

## Data Availability

The anonymized data used in this study and the R code are available on the Open Science Framework [43].
