# Supplementary material for: The Impact of a Gamified Intervention on Daily Steps in Real-Life Conditions: Retrospective Analysis of 4800 Individuals
Source: J Med Internet Res. 2024 Aug 12;26:e47116. doi: 10.2196/47116 (PMC11347891; doi:10.2196/47116)
Supplement: Multimedia Appendix 1 [file jmir_v26i1e47116_app1.pdf]

Multimedia Appendix 1: Supplemental materials

Part A. Smartphone versus wearable users' comparison.

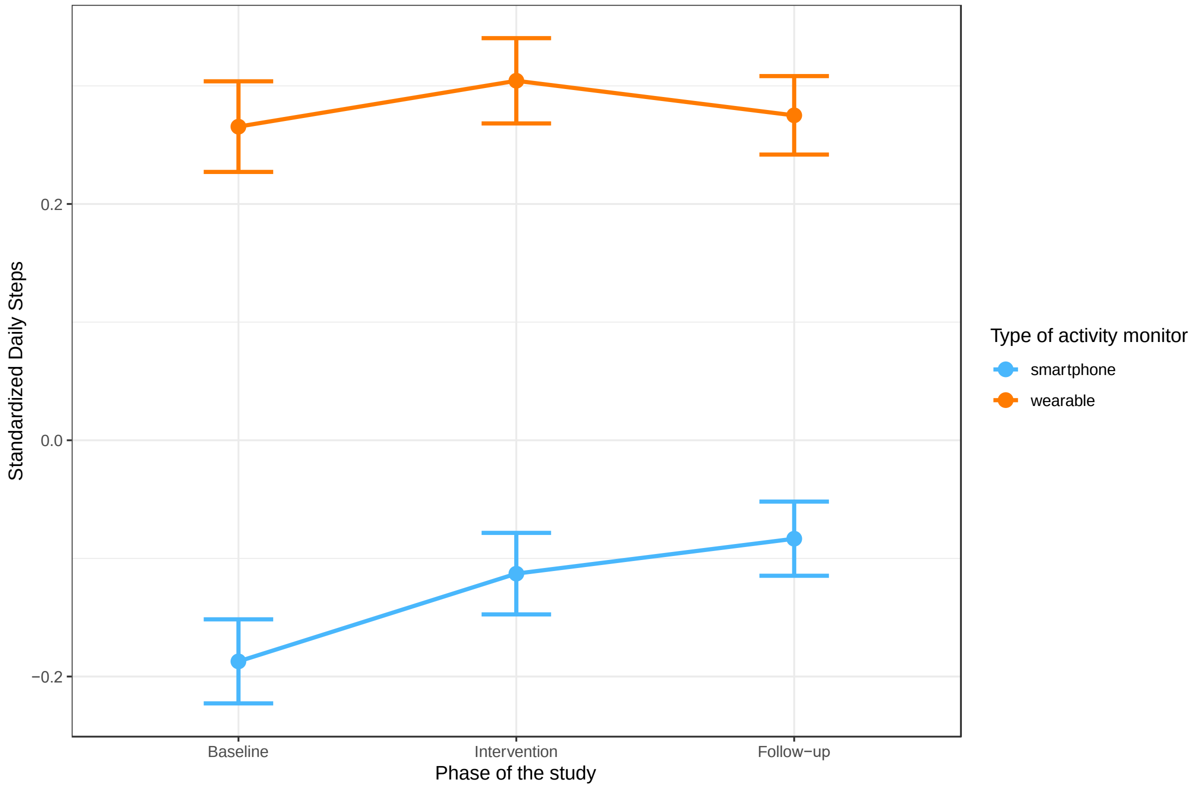

Figure S1. Changes in daily steps throughout the study phases for participants who received a Kiplin program and used their smartphone to collect their daily steps in comparison to participants who used a wearable device instead.

Part B. Analyses with outliers included

Table S1. Mixed-effects model estimates.

	Unconditional Model			Model 1			Model 2		
	b [95 CI]	SE	*P*	*b* [95 CI]	SE	*P*	*b* [95 CI]	SE	*P*
**Intercept**	-0.04 [-0.06; -0.02]	0.01	**<.001**	-0.70 [-0.78; -0.62]	0.04	**<.001**	-0.34 [-0.39; -0.29]	0.03	**<.001**
**Fixed Effects**									
Time	-0.10 [-0.11; -0.09]	0.01	**<.001**	0.11 [0.05; 0.16]	0.03	**<.001**	-0.05 [-0.06; -0.04]	0.01	**<.001**
Phases of the study									
Baseline				Reference			Reference		
Intervention				0.01 [-0.02; 0.04]	0.01	0.441	0.31 [0.28; 0.34]	0.01	**<.001**
Follow-up				-			0.29 [0.27; 0.32]	0.01	**<.001**
Participants' profile									
Age				0.01 [-0.00; 0.02]	0.01	0.117	-0.00 [-0.02; 0.01]	0.01	0.523
Sex female				Reference			Reference		
Sex male				-0.01 [-0.03; 0.01]	0.01	0.397	0.02 [-0.00; 0.05]	0.01	0.104
Condition									

Control		Reference			-		
Kiplin		-0.03 [0.07; 0.09]	0.02	0.079	-		
Population							
Workers		Reference			Reference		
Seniors		-0.05 [-0.12; 0.03]	0.04	0.213	0.13 [0.05; 0.21]	0.04	**0.002**
Obese patients		-0.12 [-0.21; -0.02]	0.05	**0.017**	-0.01 [-0.14; 0.12]	0.06	0.864
T2DM patients		-0.37 [-0.62; -0.12]	0.13	**0.003**	-0.18 [-0.44; 0.09]	0.13	0.189
Cancer patients		0.02 [-0.05; 0.09]	0.04	0.532	0.07 [-0.01; 0.15]	0.04	0.076
Other patients		-0.11 [-0.21; -0.01]	0.05	**0.031**	0.08 [-0.05; 0.21]	0.07	0.241
Type of game							
Adventure					Reference		
Boardgame					-0.17 [-0.20; -0.14]	0.02	**<.001**
Challenge					-0.08 [-0.10; -0.06]	0.01	**<.001**
Mission					-0.17 [-0.20; -0.14]	0.01	**<.001**
Exposure							
Observance ratio					0.02 [0.00; 0.04]	0.01	**0.021**
Number of games					0.02 [-0.00; 0.04]	0.01	0.064
Season							
Winter		Reference			Reference		
Spring		0.04 [0.03; 0.06]	0.01	**<.001**	0.04 [0.02; 0.05]	0.01	**<.001**
Summer		0.11 [0.09; 0.13]	0.01	**<.001**	0.06 [0.05; 0.08]	0.01	**<.001**
Fall		0.07 [0.05; 0.09]	0.01	**<.001**	0.02 [0.01; 0.03]	0.01	**<.001**
Type of device							
Android		Reference			Reference		
iOS		-0.05 [-0.08; -0.03]	0.01	**<.001**	0.02 [-0.01; 0.05]	0.01	0.128
Fitbit		0.29 [0.26; 0.33]	0.02	**<.001**	0.50 [0.47; 0.53]	0.02	**<.001**
Garmin		0.17 [0.14; 0.20]	0.02	**<.001**	0.33 [0.30; 0.36]	0.01	**<.001**
Polar		-0.94 [-2.23; -0.35]	0.66	0.154	0.11[-0.72; 0.95]	0.43	0.788
Tomtom		0.75 [0.35; 1.15]	0.21	**<.001**	0.81 [0.45; 1.18]	0.19	**<.001**
Withings		0.05 [-0.03; 0.12]	0.04	0.199	0.11 [0.05; 0.18]	0.03	**0.001**
Lockdown							
Periods without restrictions		Reference			Reference		
1st lockdown		-0.09 [-0.21; -0.01]	0.02	**<.001**	0.18 [-0.20; -0.15]	0.01	**<.001**
2nd lockdown		0.00 [-0.02; 0.03]	0.01	0.795	0.00 [-0.02; 0.02]	0.01	0.989
3rd lockdown		0.01 [-0.01; 0.03]	0.01	0.502	0.02 [0.00; 0.03]	0.01	**0.034**
**Random Effects**							
Level 1 intercept variance	0.70	0.67			0.67		
Level 2 intercept variance	0.32	1.88			0.13		
-2*log (lh)	-515610.3	-236638.1			-449086		
Akaike Information Criteria	1031232.6	473334.3			898288.4		
Marginal R^2 / Conditional R^2	0.01 / 0.38	0.071 / 0.86			0.225 / 0.39		

Notes T2DM: type 2 diabetes mellitus.

Table S2. Description of the mean daily step count during baseline, intervention, and follow-up periods, changes and relative changes from baseline in function of participants' baseline daily step count.

	**<5000**	**5000-7500**	**7501-10,000**	**>10,000**
Baseline daily step count, mean (SD)	3681 (906)	6228 (716)	8666 (718)	12,177 (1723)
Intervention daily step count, mean (SD)	8562 (4409)	10,402 (4683)	12,737 (4825)	15,670 (5290)
Follow-up daily step count, mean (SD)	5373 (2176)	6928 (1979)	8755 (2245)	11,058 (2669)
Change from baseline during the intervention	4871	4134	407	3493
Change from baseline during follow-up	1693	659	89	–1119
Relative change during intervention	+149.0 %	+66.9 %	+47.5 %	+29.8 %
Relative change during follow-up	+56.1 %	+10.9 %	1.1 %	–8.7 %

Table S3. Results of the contrast analyses performed on the mixed-effects models for each hypothesis.

	b [95 CI]	SE	*P*
**Is the gamified program effective to promote PA? (H1)**			
Overall			
Intervention | baseline	-0.16 [-0.20; -0.11]	0.02	**<.001**
Follow-up | baseline	-0.10 [-0.15; -0.05]	0.03	**<.001**
<5000			
Intervention | baseline	0.20 [0.17; 0.22]	0.01	**<.001**
Follow-up | baseline	0.17 [0.15; 0.19]	0.01	**<.001**
5000-7500			
Intervention | baseline	0.13 [0.10; 0.16]	0.01	**<.001**
Follow-up | baseline	0.10 [0.08; 0.11]	0.01	**<.001**
7501-10,000			
Intervention | baseline	0.08 [0.04; 0.12]	0.02	**<.001**
Follow-up | baseline	0.02 [0.00; 0.05]	0.03	**<.05**
>10,000			
Intervention | baseline	-0.13 [-0.18; -0.07]	0.03	**<.001**
Follow-up | baseline	-0.13 [-0.16; -0.10]	0.02	**<.001**
Smartphones vs wearable trackers			
Intervention | baseline	0.09 [0.07; 0.11]	0.01	**<.001**
Follow-up | baseline	0.04 [0.01; 0.06]	0.01	**<.001**
**Is the intervention effect greater for participants compared to nonparticipants? (H2)**			
Intervention | baseline	0.64 [0.61; 0.66]	0.01	**<.001**
**What are the moderators of the intervention effect? (H3)**			
Intervention × Age	0.07 [0.06; 0.08]	0.01	**<.001**
Intervention × Compliance ratio	0.49 [0.46; 0.54]	0.01	**<.001**
Intervention × Nb of games	-0.04 [-0.07; -0.01]	0.01	**<.001**

Table S4. Description of the mean daily step count during baseline, intervention, and follow-up periods, changes and relative changes from baseline for participants and nonparticipants.

	**Participants**	**Nonparticipants**
Baseline daily step count	7421	6574
Intervention daily step count	11,559	6581
Change from baseline during the intervention	4138.4	199.3
Relative change during intervention	+75.6 %	+9.1%

Part C. Interaction analyses for moderators of the intervention (H3)

Table S5. Interactions tested between the intervention phase, participants' characteristics, and intervention parameters in Model 2.

	b [95 CI]	SE	*P*
**Model 1**			
Intervention × Control	Reference		
Intervention × Kiplin	0.55 [0.52; 0.58]	0.02	**<.001**
**Model 2**			
Intervention × Age	0.05 [0.04; 0.06]	0.01	**<.001**
Intervention × Compliance ratio	0.37 [0.35; 0.38]	0.01	**<.001**
Intervention × Nb of games	-0.02 [-0.03; -0.00]	0.01	**0.021**
Intervention × Workers	Reference		
Intervention × Cancer	-0.18 [-0.24; -0.12]	0.03	**<.001**
Intervention × Obese	-0.07 [-0.16; 0.02]	0.04	0.133
Intervention × Senior	-0.19 [-0.25; -0.13]	0.03	**<.001**
Intervention × base <5000	Reference		
Intervention × base 5000 - 7500	-0.23 [-0.26; -0.20]	0.01	**<.001**
Intervention × base 7501 – 10,000	-0.41 [-0.44; -0.38]	0.02	**<.001**
Intervention × >10,000	-0.60 [-0.64; -0.57]	0.02	**<.001**

Notes base = baseline daily steps; control = nonparticipants

Results of contrast analyses on baseline/intervention changes between population settings:

Obese patients vs **Employees** (*b* = -0.07, 95 CI [-0.155; 0.02], *P* = 0.133)
Cancer patients vs **Employees** (*b* = -0.18, 95 CI [-0.24; -0.12], ***P* <.0001**)
Seniors vs **Employees** (*b* = -0.19, 95 CI [-0.25; -0.13], *P* **<.0001**)

—

Cancer patients vs **Obese patients** (*b* = -0.11, 95 CI [-0.21; -0.02], ***P* = 0.02**)
Seniors vs **Obese patients** (*b* = -0.12, 95 CI [-0.22; -0.02], ***P* = 0.02**)

—

Cancer patients vs **Seniors** (*b* = 0.00, 95 CI [-0.07; 0.08], *P* = 0.90)
